# Structural connectivity changes in temporal lobe epilepsy: Spatial features contribute more than topological measures

**DOI:** 10.1016/j.nicl.2015.02.004

**Published:** 2015-02-20

**Authors:** Peter N. Taylor, Cheol E. Han, Jan-Christoph Schoene-Bake, Bernd Weber, Marcus Kaiser

**Affiliations:** a Interdisciplinary Computing and Complex BioSystems (ICOS) Research Group, School of Computing Science, Newcastle University, United Kingdom; b Dept. of Biomedical Engineering, Korea University, Seoul, Republic of Korea; c Dept. of Brain and Cognitive Sciences, Seoul National University, Republic of Korea; d Center for Pediatric and Adolescent Medicine, Freiburg University, Freiburg, Germany; e Dept. of Epileptology, University of Bonn, Bonn, Germany; f Center for Economics and Neuroscience, University of Bonn, Bonn, Germany; g Institute of Neuroscience, Newcastle University, United Kingdom

**Keywords:** Epilepsy, Diffusion MRI, Brain network, Temporal lobe, Connectome

## Abstract

**Background:**

Previous studies reported reduced volumes of many brain regions for temporal lobe epilepsy (TLE). It has also been suggested that there may be widespread changes in network features of TLE patients. It is not fully understood, however, how these two observations are related.

**Methods:**

Using magnetic resonance imaging data, we perform parcellation of the brains of 22 patients with left TLE and 39 non-epileptic controls. In each parcellated region of interest (ROI) we computed the surface area and, using diffusion tensor imaging and deterministic tractography, infer the number of streamlines and their average length between each pair of connected ROIs. For comparison to previous studies, we use a connectivity ‘weight’ and investigate how ROI surface area, number of streamlines & mean streamline length contribute to such weight.

**Results:**

We find that although there are widespread significant changes in surface area and position of ROIs in patients compared to controls, the changes in connectivity are much more subtle. Significant changes in connectivity weight can be accounted for by decreased surface area and increased streamline count.

**Conclusion:**

Changes in the surface area of ROIs can be a reliable biomarker for TLE with a large influence on connectivity. However, changes in structural connectivity via white matter streamlines are more subtle with a relatively lower influence on connection weights.

## Highlights

• Using MRI data, we analyse 22 patients with left TLE and 39 non-epileptic controls.
• With a connectomics approach we investigate how nodal properties such as surface area influence connectivity weight.
• We find significant atrophy (reduced node size) in many brain areas in patients with TLE.
• We show only subtle changes in connectivity.
• When both node size and node connectivity are combined we find significant changes in connection weight.

## 1 Introduction

Epilepsy is a neurological disease characterised by abnormal electrophysiological events, leading to recurring seizures in the brain. Epileptic seizures can be broadly grouped into two categories. Generalised seizures involve widespread distributed bilateral networks, whilst focal seizures are limited to one hemisphere and involve a more localised area (Berg et al., 2010). The most common form of epilepsy is medial temporal lobe epilepsy (TLE) which most frequently occurs in the left hemisphere. Despite the traditional view of focal and generalised seizures being different in terms of their extent, recent evidence suggests involvement of brain areas far beyond the temporal lobe in TLE patients (Richardson, 2012).

Changes in grey matter volume and concentration have been shown in many brain regions in patients with TLE. Specifically, volumetric decreases have been shown in the amygdala, thalamus, entorhinal cortex, caudate nucleus, putamen and globus pallidus amongst others (DeCarli et al., 1998; Pitkänen et al., 1998; Bernasconi et al., 2004; Keller and Roberts, 2008; Meade et al., 2008). These changes are clearly wide-ranging and, although technically categorised as focal epilepsy, do involve several brain areas.

An alternative in considering brain regions on an individual basis is to consider a network of brain regions interconnected via the white matter. At the macroscopic scale, diffusion weighted magnetic resonance imaging (DW-MRI) has emerged in recent years as a valuable tool for inferring anatomical brain connectivity between brain regions (Le Bihan and Johansen-Berg, 2012; De Reus and Van den Heuvel, 2013). Some studies have found differences in TLE patient connectivity. Bonilha et al. (2012) showed a decrease in connectivity between bilateral posterior cingulate regions. Further decreases in connectivity to several other areas were also reported in the limbic network, though they were not significant after correction for false discovery rate (FDR) (Genovese et al., 2002). In a separate study by the same group patients had reduced connectivity between thalamic and precentral areas in addition to increased connectivity between parietal and supramarginal areas (Bonilha et al., 2013). In both studies the connectivity was determined as the number of streamlines between two areas, normalised by the total volume of the two areas. Another recent analysis of DW-MRI inferred connectivity in patients with left TLE also showed differences in the anatomical network of patients when compared to controls (Liu et al., 2014). That study also normalised connections between two ROIs by their average volumes. De Salvo et al. (2014) showed in patients with TLE decreases in connectivity from the cingulate, precuneus and orbitofrontal regions to other areas within the same module. In that study the connectivity weight was defined as a combination of the surface area, number of connecting streamlines and the average streamline length.

Thus, many of the previous studies of anatomical connectivity in TLE between two regions of interest (ROI) have combined (a) the number of streamlines with (b) measures influenced by the size of those ROIs (either volume or surface area) into a single value: a connection weight. Since widespread variation has been shown in the volume and surface size of many ROIs, it is unclear how each of the two measures contributes to connectivity. In this study, we systematically elucidated the changes in surface area, the changes in connectivity between areas, and their contribution to connections weights.

## 2 Methods

### 2.1 Subjects and MRI acquisition

We collected 22 left temporal lobe epilepsy subjects and 39 age-matched controls. All patients have medial temporal lobe epilepsy with unilateral hippocampal sclerosis according to MRI criteria with ipsilateral seizure onset during non-invasive/invasive EEG monitoring and underwent epilepsy surgery (selective amygdalohippocampectomy) afterwards. Further details on the subject population can be found in Table S1. For all subjects, we obtained T1-weighted MR images and diffusion-weighted MR images with a 3 Tesla scanner (Siemens MAGNETOM Trio Tim syngo, Erlangen, Germany). T1-weighted MRI data were recorded with 1 mm isovoxel, FoV 256 mm, TR = 2500 ms, and TE = 3.5 ms. DTI data were recorded with 2 mm isovoxel, FoV = 256 mm, TR = 100,000 ms, TE = 91 ms, and 64 diffusion directions with b-factor of 1000 s mm^−2^ and 12 b0 images.

We collected 22 left temporal lobe epilepsy subjects and 39 age-matched controls. All patients have medial temporal lobe epilepsy with unilateral hippocampal sclerosis according to MRI criteria with ipsilateral seizure onset during non-invasive/invasive EEG monitoring and underwent epilepsy surgery (selective amygdalohippocampectomy) afterwards. Further details on the subject population can be found in Table S1. For all subjects, we obtained T1-weighted MR images and diffusion-weighted MR images with a 3 Tesla scanner (Siemens MAGNETOM Trio Tim syngo, Erlangen, Germany). T1-weighted MRI data were recorded with 1 mm isovoxel, FoV 256 mm, TR = 2500 ms, and TE = 3.5 ms. DTI data were recorded with 2 mm isovoxel, FoV = 256 mm, TR = 100,000 ms, TE = 91 ms, and 64 diffusion directions with *b*-factor of 1000 s mm^−2^ and 12 *b*0 images.

### 2.2 Network construction

**Fig. 1:**
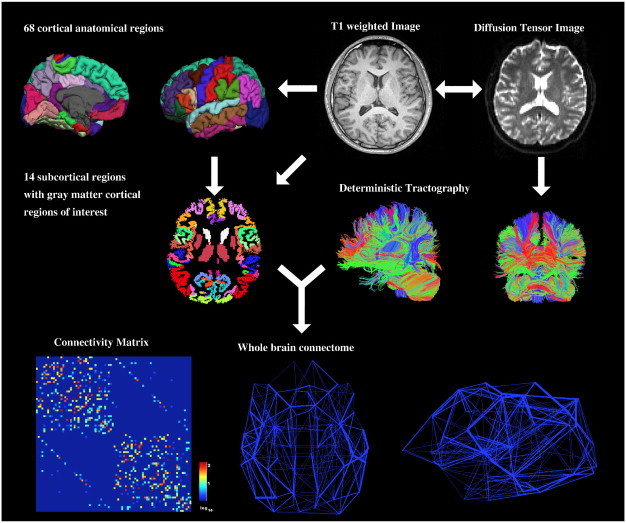
Overall procedure. From T1-weighted images, we generated 82 regions of interest (ROIs, 34 cortical areas and 7 subcortical areas a hemisphere, on the left). From diffusion weighted images, we reconstructed streamlines using deterministic tracking (on the right). Combining these two pre-processing steps, we constructed a weighted network where weights are determined by the number of streamlines connecting two ROIs.

**Fig. 2:**
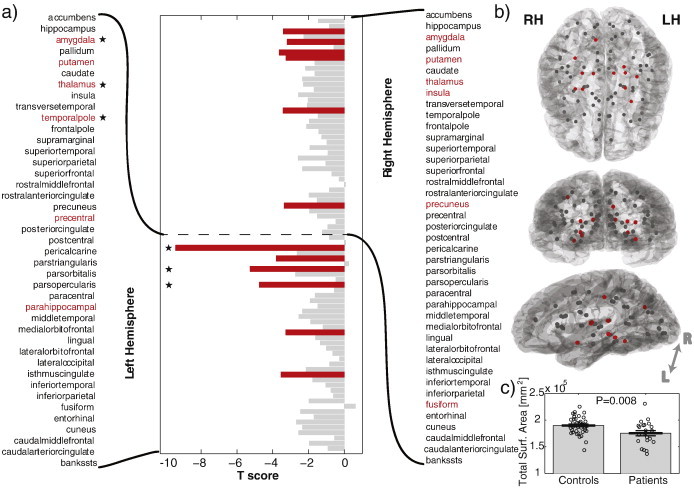
ROIs have a smaller surface area in patients than in controls. a) *t* score for surface area differences between groups. More negative values indicate a more significant decrease in patients. Significant results after FDR are indicated in red. Using a separate GLM which includes total surface area as a regressor, three areas indicated with an asterisk remain significant. b) Physical locations of the locations of the ROI used. Significant results after FDR are generally located around subcortical and left temporal areas. The projected view is shows the left hemisphere. c) The total surface area (*A^Tot^*) of all ROI is significantly decreased in patients.

We used FreeSurfer to obtain surface meshes of the boundary between grey matter and white matter from T1 anatomical brain images (http://surfer.nmr.mgh.harvard.edu, cf. Fig. 1; Image processing on the Left Side). After registering surface meshes into the diffusion space, we generated volume regions of interest (ROIs), which are voxels in the grey matter. FreeSurfer provides parcellation of anatomical regions of cortices (34 for each hemisphere) based on the Desikan atlas (Fischl et al., 2004; Desikan et al., 2006) and subcortical regions (Fischl et al., 2004, 2002) of which seven for each hemisphere (nucleus accumbens, amygdala, caudate, hippocampus, pallidum, putamen, and thalamus) were included (see Fig. 2a for names of ROIs). Thus, our structural brain networks, observing both hemispheres, consisted of 82 cortical and subcortical regions in total. Parcellation and registration were manually checked for errors by visual inspection.

To obtain streamline tractography from eddy-current corrected diffusion tensor images (FSL, http://www.fmrib.ox.ac.uk/fsl/), we used the Fiber Assignment by Continuous Tracking (FACT) algorithm (Mori and Barker, 1999) with 35° of angular threshold through diffusion toolkit along with TrackVis (Wang et al., 2007) (Fig. 1 image processing on the right side).

For network reconstruction, we modified the Ucla Multimodal Connectivity Package (UMCP, http://ccn.ucla.edu/wiki/index.php) to obtain connectivity matrices from the defined and registered ROIs and tractography. We used the number of connecting streamlines to determine our connectivity matrix (*M*). We also computed the Euclidean distance between the centre of each ROI to give us a distance network *E*. To compute the surface areas of each ROI (A_i_) FreeSurfer was used for cortical ROIs, and we computed the interface area to white matter in T1 space for subcortical ROIs, whilst Zhang et al. (2011) and Liao et al. (2010) computed interface areas to white matter in DTI space for all ROIs. We also computed a streamline length matrix *L* in which each element *L*_*i*,*j*_ corresponds to the mean length of the streamlines connecting ROI *i* to ROI *j* in millimetres (mm). For connections that are absent, the streamline length is set to zero and disregarded from the statistical analysis (see further details; Section 2.3). Finally, to aid comparison with previous studies we defined a connection weight. The weight between ROI *i* and ROI *j* is defined as:

$$W_{i,j}=\left(\frac{2}{A_{i}+A_{j}}\right)\times M_{i,j}\times \left(\frac{1}{L_{i,j}}\right)$$

And is similar to that defined by Hagmann et al. (2008). Our data for analysis is therefore as specified in Table 1.

### 2.3 Statistical tests & visualisation

All topological and statistical operations were conducted using MATLAB (Version 2012a, MathWorks, Natick, USA). We used a general linear model (GLM) to regress age and gender and to find group differences (fitlm method in Matlab). We also, where stated, include total brain surface area (*A^Tot^*) as a regressor to check if results can be attributed to global brain reduction, or if they are over and above such reductions. We define total surface area as *A^Tot^* = Σ^*i* = 1…82^*A_i_* in the 82 node network. Where mentioned, false discovery rate (FDR) was applied with a 5% significance level using custom code to correct for multiple comparisons. In group analysis of *L*, *M*, and *W* we only included elements where a connection was present for the majority of subjects in one or more of the subject groups to ensure that a genuine difference was observed. We also used partial least squares regression to account for multicolinearities in the data (Supplementary Fig. S4) to measure the contribution of *M_i,j_*, *S_i,j_*, *Li*,*j* and *A^Tot^* to *Wi*,*j* since various factors can be correlated and act as a predictor (Lefebvre et al., 2014). To test for normality of distributions we use the Lilliefors test. To test for differences between groups (patients & controls) the group was included as a categorical variable in the model and the *t*-statistic and *p*-value was calculated, which tests for the significance of that term in the model. For visualisation, a standard template brain mesh was overlaid with the average ROI coordinates for all subjects (Collins et al., 1998).

All topological and statistical operations were conducted using MATLAB (Version 2012a, MathWorks, Natick, USA). We used a general linear model (GLM) to regress age and gender and to find group differences (fitlm method in Matlab). We also, where stated, include total brain surface area (*A^Tot^*) as a regressor to check if results can be attributed to global brain reduction, or if they are over and above such reductions. We define total surface area as *A^Tot^* = Σ_*i* = 1…82_*A_i_* in the 82 node network. Where mentioned, false discovery rate (FDR) was applied with a 5% significance level using custom code to correct for multiple comparisons. In group analysis of *L*, *M*, and *W* we only included elements where a connection was present for the majority of subjects in one or more of the subject groups to ensure that a genuine difference was observed. We also used partial least squares regression to account for multicolinearities in the data (Supplementary Fig. S4) to measure the contribution of *M*_*i*,*j*_, *S*_*i*,*j*_, *L*_*i*,*j*_ and *A^Tot^* to *W*_*i*,*j*_ since various factors can be correlated and act as a predictor (Lefebvre et al., 2014). To test for normality of distributions we use the Lilliefors test. To test for differences between groups (patients & controls) the group was included as a categorical variable in the model and the *t*-statistic and *p*-value was calculated, which tests for the significance of that term in the model. For visualisation, a standard template brain mesh was overlaid with the average ROI coordinates for all subjects (Collins et al., 1998).

## 3 Results

The results are presented in five sections. First, we investigate changes in the surface area of ROIs. Second, we show differences in the position of ROIs, relative to each other (i.e. the Euclidean distance between the centre of them). Third, we then investigate changes in the number of connecting streamlines between ROIs. Fourth, we observe changes in the mean length of connecting streamlines. Note that this mean length follows the actual three-dimensional trajectory of a fibre tract and, depending on the degree of fibre curvature, can strongly deviate from the Euclidean distance between the connected ROIs. Finally we investigate how these changes jointly contribute to changes observed in connection weight.

### 3.1 ROIs in patients have a smaller surface area than in controls

The surface area of the majority of ROIs in the network was decreased in patients, relative to controls. Fig. 2 shows the *t*-score for each ROI. More negative values indicate a smaller surface area for patients, relative to controls, for that ROI. Eleven of the 82 ROIs survived FDR (indicated in red), the majority of which were located in the left hemisphere and in subcortical structures of the right hemisphere. Overall there is a clear global reduction in total surface area in patients (Fig. 2c). When including the total surface area as a regressor three ROIs remain significant (*p* < 0.0001) with their reductions in surface area over and above the global decreases. These areas are indicated by asterisks on Fig. 2a.

### 3.2 ROIs are physically closer to each other in patients

**Fig. 3:**
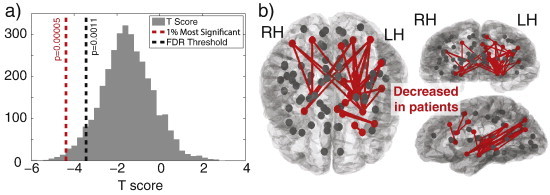
Most ROIs are closer together in patients than in controls. a) Distribution of *t* scores representing changes in Euclidean distance between ROIs. More negative values indicate a decrease in Euclidean distance between two ROIs in patients (bin number = 35). Distances between ROIs with *t* < −4.3 have a *p* < 0.0001 and represent the 1% most significant changes. b) The most significant decreases in Euclidean distance between ROIs in patients are located predominantly in the left hemisphere and many involving subcortical areas. A decrease between two ROIs is indicated by a red line between those ROIs.

Logically, if the surface area of two adjacent parcellated regions decreases equally in all directions, the Euclidean distance between the centre of those ROI will also decrease. In Fig. 3a we show the distribution of *t*-scores of Euclidian distances between all ROIs. As expected with such widespread changes in surface area (Fig. 2), the entire distribution is centred around a *t*-score of −1.7, with only very few (statistically insignificant) ROIs further apart in patients. This means that the vast majority of distances between ROI are decreased in patients, since the vast majority have negative *t*-scores. The *t*-score can, in effect be considered a measure of shrinkage between two ROIs. Since the changes are so widespread many results remain after FDR correction.

In Fig. 3b we show the top 1% most significant changes in Euclidean distance between ROI. These are generally located in the left hemisphere in the subcortical, temporal and parietal areas and represents those with *t*-scores of less than −4.3 (*p* < 0.0001).

These decreases within the left hemisphere are also consistent if the total brain surface area is included as a further regressor (Supplementary Fig. S1). Several distances are decreased in patients over and above what would be expected, given the global brain decreases in surface area. These are significant after FDR correction.

These decreases within the left hemisphere are also consistent if the total brain surface area is included as a further regressor (Supplementary Fig. S1). Several distances are decreased in patients over and above what would be expected, given the global brain decreases in surface area. These are significant after FDR correction.

### 3.3 Subtle changes in connectivity

Straightforward analysis of the number of streamlines (*M*) revealed no significant differences after FDR correction. With *t*-scores on average around zero, the networks are seemingly similar in patients and controls (Fig. S2a). Indeed, the 10% most significant differences (*p* < 0.041, |*t*| > 2.1) appear to be fairly evenly and randomly distributed throughout the brain with no obvious spatial profile as in the previous analysis (for example, Figs. 2 and 3, which are predominantly in the left hemisphere). The spatial locations of these decreases/increases in the number of streamlines are shown in red/blue, respectively, in Supplementary Fig. S2b. If total brain surface area is included as a regressor there is a significant increase in connectivity between the insular and superior temporal cortex areas in the left hemisphere (*p* < 0.0001).

Straightforward analysis of the number of streamlines (*M*) revealed no significant differences after FDR correction. With *t*-scores on average around zero, the networks are seemingly similar in patients and controls (Fig. S2a). Indeed, the 10% most significant differences (*p* < 0.041, |*t*| > 2.1) appear to be fairly evenly and randomly distributed throughout the brain with no obvious spatial profile as in the previous analysis (for example, Figs. 2 and 3, which are predominantly in the left hemisphere). The spatial locations of these decreases/increases in the number of streamlines are shown in red/blue, respectively, in Supplementary Fig. S2b. If total brain surface area is included as a regressor there is a significant increase in connectivity between the insular and superior temporal cortex areas in the left hemisphere (*p* < 0.0001).

### 3.4 Subtle changes in streamline length

We find no significant differences in mean streamline length between ROIs. Supplementary Fig. S3a shows the distribution of *t*-scores (negative values indicate a decrease in patients). With a mean of approximately zero there is no clear skew as in Fig. 3a. Furthermore, there are no specific obvious spatial structures when considering the most different streamline lengths (top 10% most different shown in Supplementary Fig. S3b). This is also the case when including the total brain surface area as a regressor, with no significant differences or obvious spatial profile (result not shown).

We find no significant differences in mean streamline length between ROIs. Supplementary Fig. S3a shows the distribution of *t*-scores (negative values indicate a decrease in patients). With a mean of approximately zero there is no clear skew as in Fig. 3a. Furthermore, there are no specific obvious spatial structures when considering the most different streamline lengths (top 10% most different shown in Supplementary Fig. S3b). This is also the case when including the total brain surface area as a regressor, with no significant differences or obvious spatial profile (result not shown).

### 3.5 Significant changes in connection weight

**Fig. 4:**
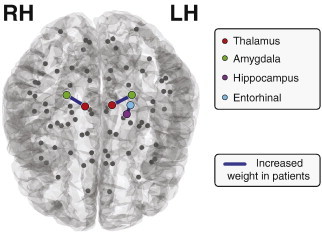
Significant changes in connection weight. Three connections have increased connection weight in patients (*p* < 0.0015) significant after FDR correction.

Upon investigation of the weight based measure (W) we find three connections significantly different after FDR in patients. All three have increased weight in patients relative to controls. These are shown topographically in Fig. 4 and include bilateral thalamic–amygdala connections, in addition to increased hippocampal-entorhinal connectivity weight. When including total surface area in the GLM only the bilateral thalamus–amygdala connections remain significantly different, suggesting these changes in weight are not accounted for by total surface area and thus highly significant.

Since three factors contribute to connectivity weight (W) it is unclear, by considering weight alone, which factors influence the measured differences between groups, and by how much (to see how each measure contributes to the connectivity weight itself, rather than the difference see Supplementary Fig. S4). In Table 2 we investigate how this relationship unfolds by showing the *t*-scores for the three connections in question and for the three factors which contribute to it.

Since three factors contribute to connectivity weight (W) it is unclear, by considering weight alone, which factors influence the measured differences between groups, and by how much (to see how each measure contributes to the connectivity weight itself, rather than the difference see Supplementary Fig. S4). In Table 2 we investigate how this relationship unfolds by showing the *t*-scores for the three connections in question and for the three factors which contribute to it.

For all three connections, the surface area difference is large and therefore contributes to the difference in weight. This can be specifically seen for the connection between the left thalamus and left amygdala, where, although the number of streamlines (*t* = 1.52) and the mean streamline length (*t* = −0.53) are similar between groups, the difference in surface area is so large & significant (*t* = 7.63) that the weight becomes significant too. Interestingly, the same connection in the right hemisphere has a significant difference in weight, however, this is not due solely to surface area differences but rather a combination of surface area and number of streamlines. Connectivity between the hippocampus and entorhinal cortex is more influenced by an increase in the number of streamlines at the same time as a decrease in surface area, whilst the streamline length plays a less influential role.

## 4 Discussion

In this study we have investigated differences in brain network features between patients with left temporal lobe epilepsy and nonepileptic controls. In patients, we found significant decreases in the surface area of ROIs and the Euclidian distance between them. In contrast, we found only subtle differences in the number of connecting streamlines between ROIs and mean streamline length. Finally we showed increases in connectivity weight in patients which can be mainly explained by alterations in ROI surface area and increases in streamline number. The subtle increase in streamline number, when combined with large decreases in surface area then becomes significant.

Many previous studies demonstrating changes in patient connectivity have used a measure of connection weight which incorporates at least two different factors (Bonilha et al., 2012, 2013; De Salvo et al., 2014; Liu et al., 2014). First of all a measure of the number of streamlines/fibres is used (either probabilistic or deterministic). Secondly the volume/surface area of the two connecting ROIs is used. Since so many ROIs have decreased surface area it is not surprising that alterations in connectivity have been observed in those studies which consequently impacts graph theoretic measures (Kaiser, 2011).

Our observation of widespread reduction in ROI surface area is in accordance with many early MRI studies describing atrophy of brain regions in patients with TLE (DeCarli et al., 1998; Pitkänen et al., 1998; Bernasconi et al., 2004; Keller and Roberts, 2008; Meade et al., 2008; Bonilha et al., 2010). However, in our approach we have categorically computed the surface area of all ROIs, rather than focusing on specific individual areas. Indeed some areas, such as the ipsilateral amygdala, are drastically reduced in patients as is perhaps expected following previous work (Pitkänen et al., 1998; Kullmann, 2011), however, what is interesting is just how widespread the decreases in surface area are. The decreases can be observed far beyond the temporal lobe.

Such widespread decreases in ROI surface area intuitively leads to the suggestion that the ROI are more proximal. Indeed, we found this to be the case in our analysis. This was most significant in the left hemisphere (ipsilateral to the epileptic focus). A possible reason for this implication in epilepsy could be that pathological spreading has less distance to travel (e.g. using short-range connections through the grey matter) in order to recruit more tissue when there is a decrease in distance between ROI. The resulting reduced transmission delays in propagating activity could facilitate synchronous pre-synaptic neurotransmitter release and consequently increase post-synaptic activity leading to increased population activity. This could be tested in the future using, for example, a computational model of spreading (Kaiser et al., 2007; Kaiser, 2013; Taylor et al., 2013). Whilst in this study we determined the proximity of regions by calculating the Euclidean distance, a future study might use geodesic distance as this is directly biologically interpretable as lateral connectivity within the grey matter. A surprising finding is that although ROIs are more closely located, the average lengths of the connecting streamlines between them are similar. To our knowledge this is the first time that this has been reported in a whole brain scale analysis of TLE. A possible explanation for this could lie in the fact that an inherent problem with DTI tracking algorithms is their propensity to favour shorter, straighter streamlines (Jones, 2010). It could be that the longer, more curved fibres are present in the controls but simply not detected by the algorithm. Alternatively, this might indicate that reductions in grey matter volume in patients occur after fibre tracts between ROIs have been established in an individual.

Another, perhaps unexpected, finding is that the number of streamlines between ROIs is broadly similar between groups. This is despite several other studies finding changes in network connectivity (Bonilha et al., 2012, 2013; De Salvo et al., 2014; Liu et al., 2014). However, in those studies the connecting number of streamlines was either adjusted by the volume or surface area of the connected ROIs, the length of the connecting streamlines, or a combination of all three. Since widespread atrophy is well known in TLE we chose to investigate each aspect separately. Essentially our results suggest that abnormal (atrophied) nodes contribute to seizure initiation, but that large scale seizure spreading in the patient (e.g. secondary generalisation — if any) may occur through otherwise normal connectivity through the white matter.

In the present study we did not investigate the role of functional connectivity and focused only on structural brain properties. It has been shown in several studies that alterations in functional correlations between brain regions exist in patients with both generalised (Zhang et al., 2011; Masterton et al., 2012; McGill et al., 2012) and focal seizures including TLE (Liao et al., 2010, 2011; Pittau et al., 2012). How structural factors such as alterations in the surface area of nodes impacts the fMRI correlation between them is not fully understood (Taylor et al., 2014).

Another potential limitation of this study is that we used the FACT algorithm which is unable to resolve crossing fibres due to our use of DTI as opposed to DSI or HARDI analysis (Jbabdi and Johansen-Berg, 2011). This probably leads to an underestimation of the connectivity and thus may contribute to our inability to detect statistically significant differences between groups for our number of streamlines analysis. Furthermore, we used deterministic tractography as opposed to probabilistic tractography which was used in some of the other DTI studies of TLE (Bonilha et al., 2012, 2013). This may potentially explain why those studies found slightly different results in terms of individual connections. Another measure of connectivity is mean fractional anisotropy (FA) along the streamlines which connect ROIs. Besson et al. (2014) recently used probabilistic tractography in conjunction with mean FA and showed decreases in left hemispheric connectivity in patients. It is unclear what contributed to the decreases in connectivity in that study (i.e. FA, streamline number or both). It is clear, however, that those connections identified in that study clearly overlap with the atrophied regions in ours (cf. their figures 3a & 5a with our Figs. 2b and 3b) suggesting the importance of regional atrophy in considering wider connectivity.

An interesting aspect of the study by Besson et al. (2014) is that they studied patients with right TLE in addition to left TLE. They found differences to controls which were not symmetric with the alterations in patients with left TLE, compared to controls. This means that the approach of Bonilha et al. (2013), which involves flipping the right and left hemispheres in the connectivity matrix to combine all subjects together in one group, may lead to several more diffuse abnormal connections appearing which are not actually present in the left TLE group alone. This could therefore also explain some of the differences in abnormal connections found in that study and ours.

Due to metadata being unavailable we were not able to investigate the potential impact of seizure duration on the structural brain properties studied here. This could be important as a previous study has shown that cortical thinning occurs in patients with epilepsy and that this is correlated with seizure duration (Bernhardt et al., 2010). Furthermore, reductions in hippocampal volume are correlated with seizure duration (Jokeit et al., 1999; Theodore et al., 1999) in TLE patients. It is therefore reasonable to suggest some of our analysis may be impacted by this which, if included, would give greater statistical power.

In conclusion, we suggest that caution should be exercised when interpreting results which incorporate factors such as grey matter volume or surface area into white matter connectivity matrices. This is also the case for connectomics studies of other diseases where atrophy is known to play a role such as for schizophrenia (Vita et al., 1988) and Alzheimer's disease (Chan et al., 2001). Disentangling the relationship between abnormal node properties and abnormal networks will be a challenge for future studies. We therefore suggest to carefully observe the contributions to connection weights of spatial features such as region size/volume, fibre trajectory length, and Euclidean distance between regions. Indeed, how one defines connectivity will certainly impact the results, and consequently the interpretation.

The following are the supplementary data related to this article.

Supplementary Fig. 1 The Euclidean distance between some ROIs is significantly different between patients and controls when including total brain surface area (*A^Tot^*) as a regressor.

Supplementary Fig. 2 The number of connecting streamlines between ROIs is not significantly different in patients. a) Distribution of nonzero *t*-scores for the number of connecting streamlines between all ROIs (bin number = 35). None remain as significant after FDR correction. The 1% most significant lie to the extremities beyond the dashed line *t*-scores are normally distributed (*p* = 0.5). b) Spatial arrangement of the 1% most different number of connecting streamlines in patients. Blue represents an increase, whilst red indicates decrease in patients.

Supplementary Fig. 3 The mean streamline lengths (in mm) between ROIs are not significantly different in patients. a) Histogram of *t*-scores indicative of differences between groups. Negative values indicate a decrease in patients. *t*-Scores are not normally distributed (*p* = 0.04) b) the 1% most different between patients and controls (*p* < 0.043, |*t*| > 1.9). None are significant after FDR correction.

Supplementary Fig. 4 Contribution of ROI surface area, number of streamlines, mean streamline length and total surface area of the brain to the connection weight. For methods see supplementary text S1.

Table S1 Subject data.

Supplementary data related to this article can be found online at http://doi.dx.org/10.1016/j.nicl.2015.02.004.

## Figures and Tables

**Table 1:** Analysed network features where *i* and *j* represent different ROIs.

Measure	Interpretation
*M*_*i*,*j*_	Number of connecting streamlines between *i* and *j*
*E*_*i*,*j*_	Euclidean distance between *i* and *j* [mm]
*L*_*i*,*j*_	Mean spatial length of connecting streamlines between *i* and *j* [mm]
*W*_*i*,*j*_	Weight of connection between *i* and *j*
*A_i_*	Surface area of *i* [mm^2^]

**Table 2:** *t*-Score showing differences between patients & controls. Positive values indicate an increase in patients. For surface area the following formula was used $\frac{2}{A_{i}+A_{j}}$ where *A_i_* and *A_j_* represents the surface area of ROI *i* and *j* respectively. SL stands for streamline. Results significant after FDR correction are indicated with asterisks. Results in the top 10% most significant are indicated with a +.

Connection	Weight	SL num	Surface area	SL length
L. thalamus–L. amygdala	4.3285+*	1.5185	7.6372+*	−0.52774
L. entorhinal–L. hippocampus	4.0986+*	2.6754+	2.084	−0.43003
R. thalamus–R. amygdala	4.0682+*	2.3778+	3.6158+*	0.80549
