# Supplementary material for: Structural connectivity changes in temporal lobe epilepsy: Spatial features contribute more than topological measures
Source: Neuroimage Clin. 2015 Feb 20;8:322–8. doi: 10.1016/j.nicl.2015.02.004 (PMC4473265; doi:10.1016/j.nicl.2015.02.004)

# Patient / control differences in Euclidean distance between ROI when total brain surface area is included as a regressor.

Significant after FDR (p<0.00015)

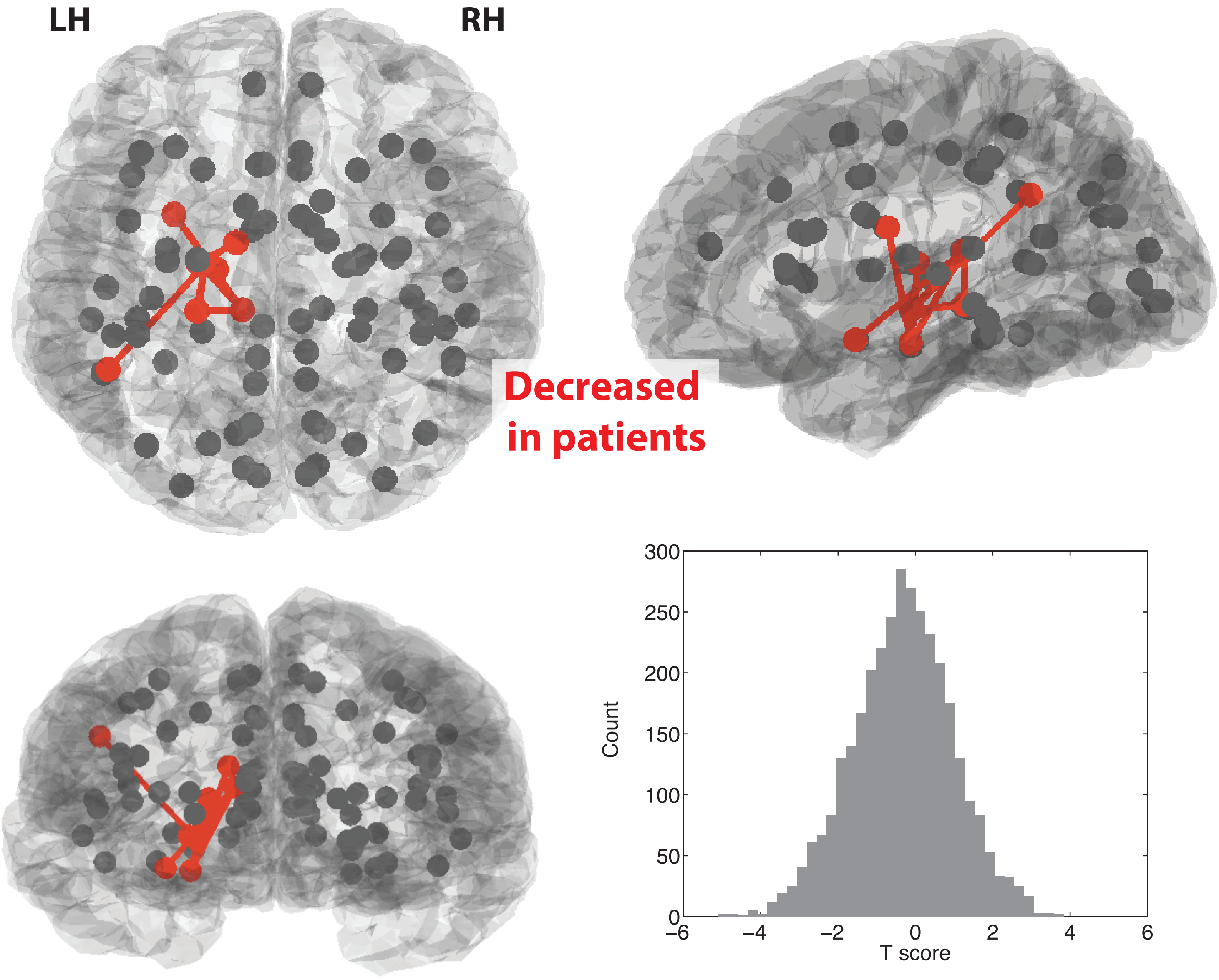

Supplement: Supplementary Fig. 1 — The Euclidean distance between some ROIs is significantly different between patients and controls when including total brain surface area (ATot) as a regressor. [file mmc1.pdf]
