# Supplementary figures and images for: Structural connectivity changes in temporal lobe epilepsy: Spatial features contribute more than topological measures
Source: Neuroimage Clin. 2015 Feb 20;8:322–8. doi: 10.1016/j.nicl.2015.02.004 (PMC4473265; doi:10.1016/j.nicl.2015.02.004)

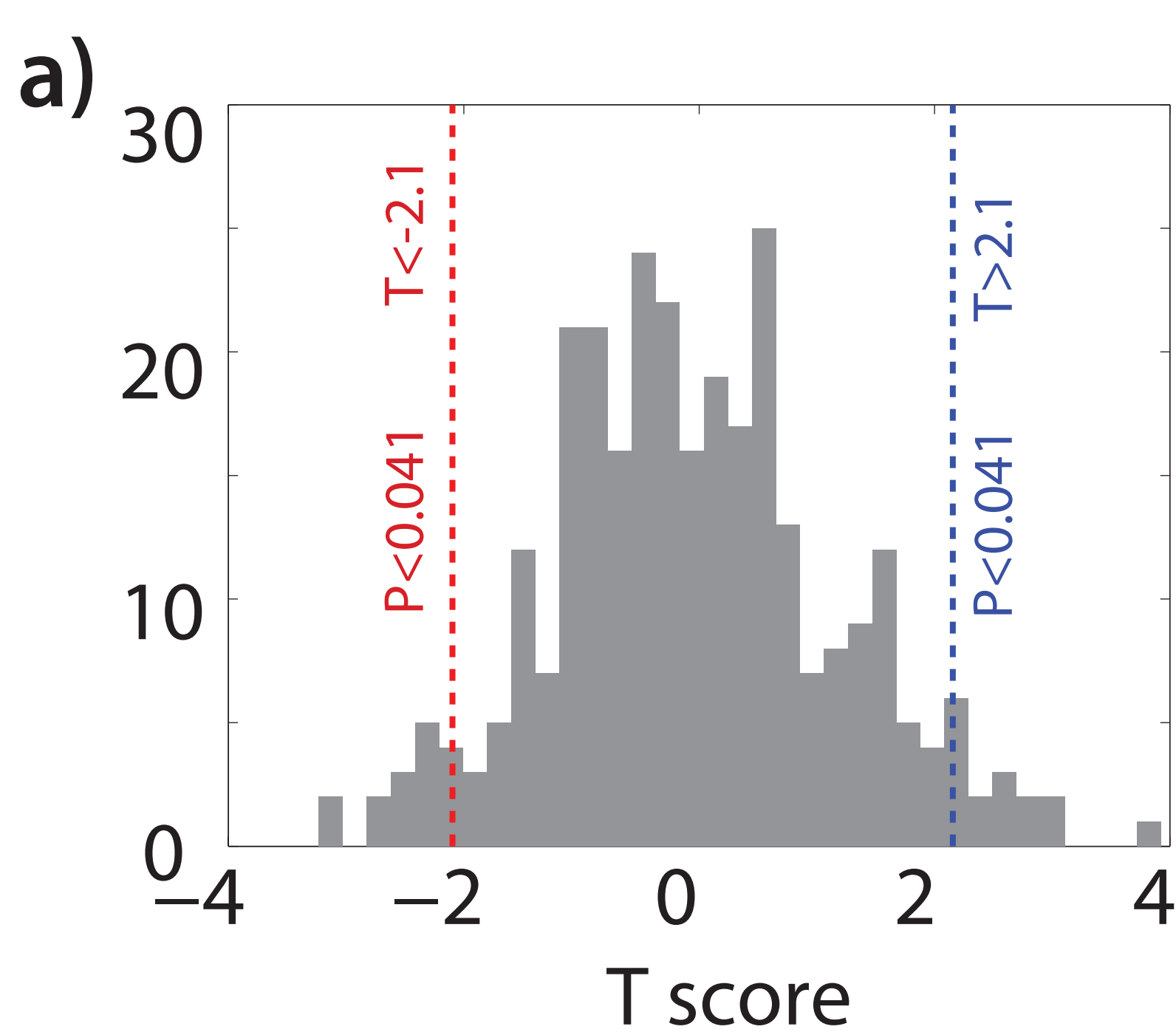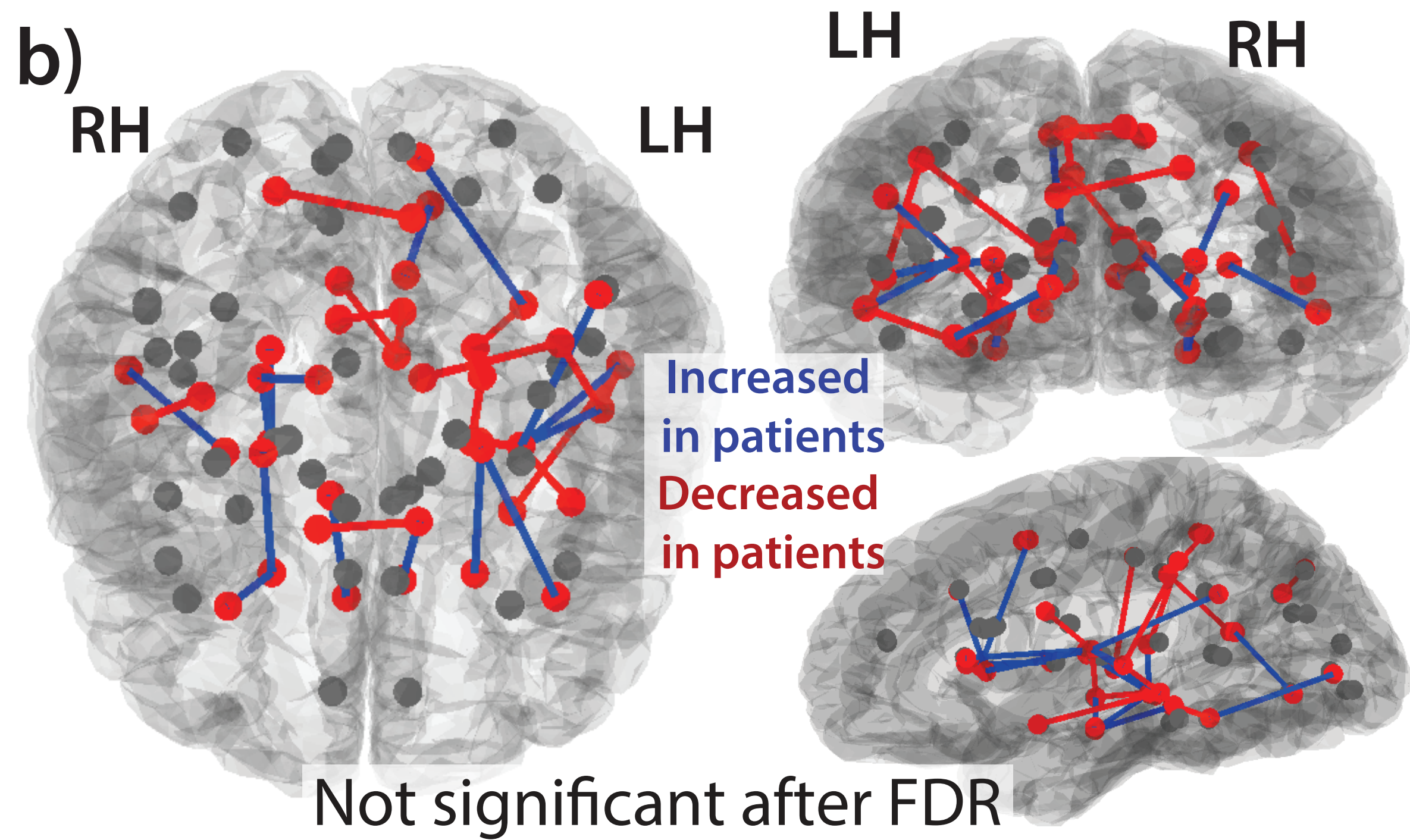

Supplement: Supplementary Fig. 2 — The number of connecting streamlines between ROIs is not significantly different in patients. a) Distribution of nonzero t-scores for the number of connecting streamlines between all ROIs (bin number = 35). None remain as significant after FDR correction. The 1% most significant lie to the extremities beyond the dashed line t-scores are normally distributed (p = 0.5). b) Spatial arrangement of the 1% most different number of connecting streamlines in patients. Blue represents an increase, whilst red indicates decrease in patients. [file mmc2.pdf]

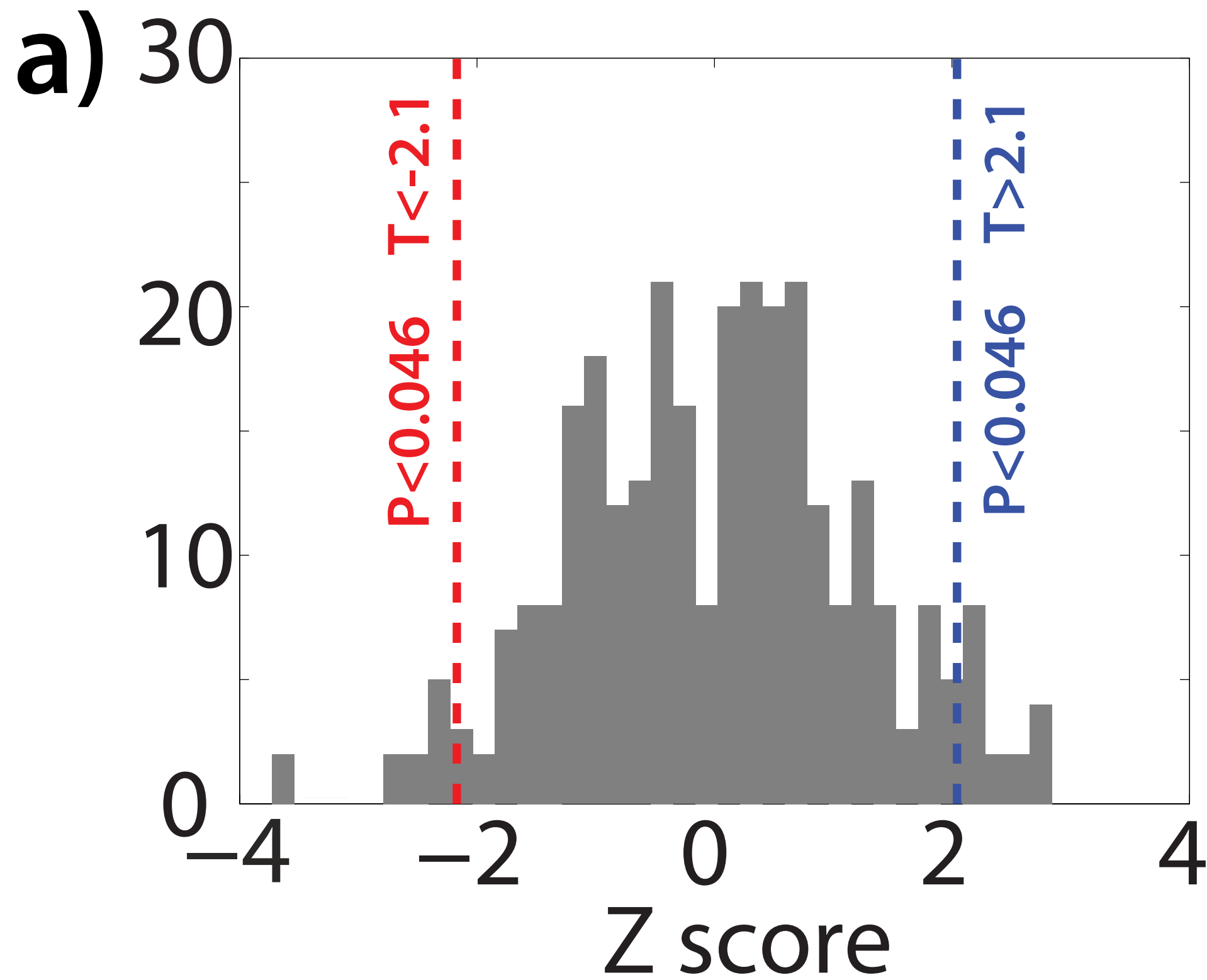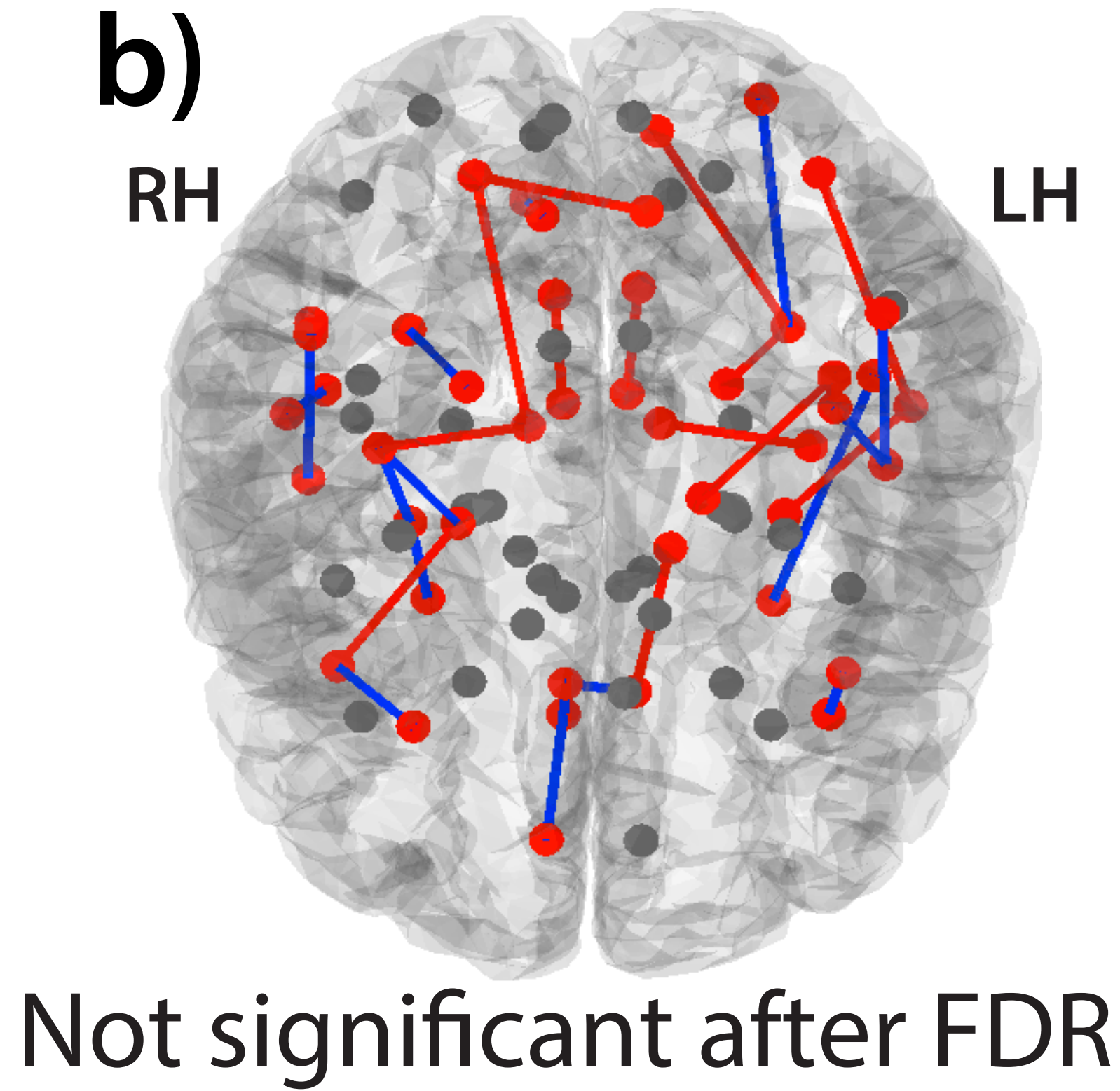

Supplement: Supplementary Fig. 3 — The mean streamline lengths (in mm) between ROIs are not significantly different in patients. a) Histogram of t-scores indicative of differences between groups. Negative values indicate a decrease in patients. t-Scores are not normally distributed (p = 0.04) b) the 1% most different between patients and controls (p < 0.043, |t| > 1.9). None are significant after FDR correction. [file mmc3.pdf]
