# Supplementary material for: Structural connectivity changes in temporal lobe epilepsy: Spatial features contribute more than topological measures
Source: Neuroimage Clin. 2015 Feb 20;8:322–8. doi: 10.1016/j.nicl.2015.02.004 (PMC4473265; doi:10.1016/j.nicl.2015.02.004)
Supplement: Supplementary Fig. 4 — Contribution of ROI surface area, number of streamlines, mean streamline length and total surface area of the brain to the connection weight. For methods see supplementary text S1. [file mmc4.docx]

**Supplementary Figure S4**

Two connections have significantly different weights between patients and controls when accounting for age, gender and total surface area in the regression. However, several parameters might influence differences between subject groups. In the following we investigate how the different factors which compose the weight measure (ROI surface area (ROI SA), number of streamlines, and mean streamline length) contribute to its value in addition to the total surface area of the brain.

Partial least squares regression (PLS), which accounts for multicollinearities in the data, produces a beta value for each of the regressors (in this case four shown on the x axis). Here, the beta value is effectively the gradient of a line of best fit through the data for each variable following the normalization of each variable (mean subtracted, divided by standard deviation). A perfect fit (prediction) would have a beta value of 1 or -1, whereas no fit would be zero. In the following we show the beta values for each variable as a proportion of the sum of the absolute value of all beta values. This is termed the beta contribution. As an example, if all four variables contribute the same amount to the prediction of the connectivity weight, then all would have a beta contribution of ±0.25 (i.e. each contributes 25% to the PLS prediction). Thus, values larger in magnitude contribute more to the weight. Negative values contribute by a negative correlation (i.e. *shorter* streamlines - SL Len - have a *stronger* weight). This is only valid given that the PLS model provides a good prediction of the data, given the variables of interest. Indeed, this is the case with over 90% prediction accuracy (this high accuracy is not surprising, given that the weight is composed of three of the predictors).

The result shows that the connectivity weight is determined mainly by the number of streamlines, the mean streamline length and, to a lesser extent the ROI surface area. The total brain surface area contributes little to improving the prediction of the weight. The general trend of contributions is consistent in both patients and controls since both are normalized (predictors’ mean subtracted, divided by standard deviation) separately in this particular analysis.


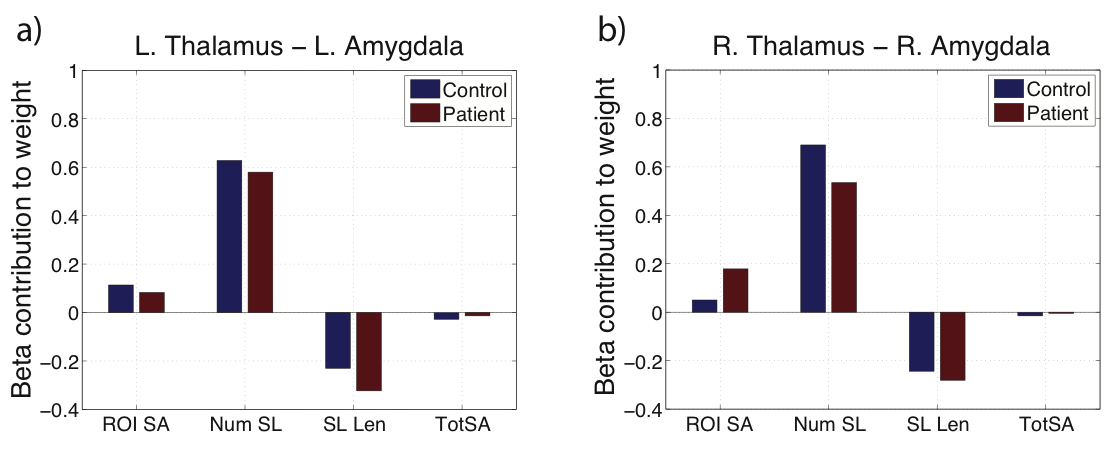


Reference

1 de Jong, S. "SIMPLS: An Alternative Approach to Partial Least Squares Regression." *Chemometrics and Intelligent Laboratory Systems*. Vol. 18, 1993, pp. 251–263.

2 Rosipal, R., and N. Kramer. "Overview and Recent Advances in Partial Least Squares." *Subspace, Latent Structure and Feature Selection: Statistical and Optimization Perspectives Workshop (SLSFS 2005), Revised Selected Papers (Lecture Notes in Computer Science 3940)*. Berlin, Germany: Springer-Verlag, 2006, pp. 34–51.
