# Supplementary material for: Structural connectivity changes in temporal lobe epilepsy: Spatial features contribute more than topological measures
Source: Neuroimage Clin. 2015 Feb 20;8:322–8. doi: 10.1016/j.nicl.2015.02.004 (PMC4473265; doi:10.1016/j.nicl.2015.02.004)
Supplement: Table S1 — Subject data. [file mmc5.docx]

**Table S1:** Subject data

| **Patients** | | **Controls** | |
| --- | --- | --- | --- |
| **Age** | **Gender** | **Age** | **Gender** |
| 22 | M | 18 | F |
| 23 | F | 19 | M |
| 24 | F | 21 | M |
| 27 | M | 22 | M |
| 29 | F | 23 | F |
| 32 | M | 24 | F |
| 32 | F | 24 | F |
| 33 | F | 26 | M |
| 39 | M | 27 | M |
| 39 | F | 31 | F |
| 43 | F | 32 | M |
| 44 | F | 33 | F |
| 44 | M | 34 | M |
| 45 | F | 35 | F |
| 45 | F | 36 | M |
| 46 | M | 37 | M |
| 48 | F | 39 | M |
| 49 | M | 39 | F |
| 54 | F | 41 | M |
| 54 | F | 42 | F |
| 62 | F | 44 | M |
| 68 | F | 45 | M |
|  |  | 45 | F |
|  |  | 45 | F |
|  |  | 46 | M |
|  |  | 46 | F |
|  |  | 46 | M |
|  |  | 47 | M |
|  |  | 48 | F |
|  |  | 48 | F |
|  |  | 49 | M |
|  |  | 50 | F |
|  |  | 53 | M |
|  |  | 54 | F |
|  |  | 55 | F |
|  |  | 58 | F |
|  |  | 61 | F |
|  |  | 67 | F |
|  |  | 70 | F |
